# Tumour purity as an underlying key factor in tumour mutation detection in colorectal cancer

**DOI:** 10.1002/ctm2.1252

**Published:** 2023-05-02

**Authors:** Tao Yu, Qianpeng Huang, Xinyu Zhao, Shiyao Zhang, Qi Zhang, Xingcan Fan, Gang Liu

**Affiliations:** ^1^ Department of Oncology Tianjin Medical University General Hospital Tianjin China; ^2^ Department of General Surgery Tianjin Medical University General Hospital Tianjin China; ^3^ Department of General Surgery Handan Central Hospital Handan China

## Dear Editor

The emergence of next‐generation sequencing (NGS) technology has enabled the large‐scale identification of personalised genetic characteristics of colorectal cancer (CRC).^1^ However, the accuracy may be influenced by certain sample factors, such as sampling methods, biospecimen type (fresh vs. formalin‐fixed paraffin‐embedded) and input DNA amount.^2^, ^3^, ^4^ We creatively performed a contrastive analysis based on homogenous paired real‐world surgical tumour specimens to comprehensively assess the impact of low tumour cell fraction on the authenticity of somatic mutation calling.

**FIGURE 1:**
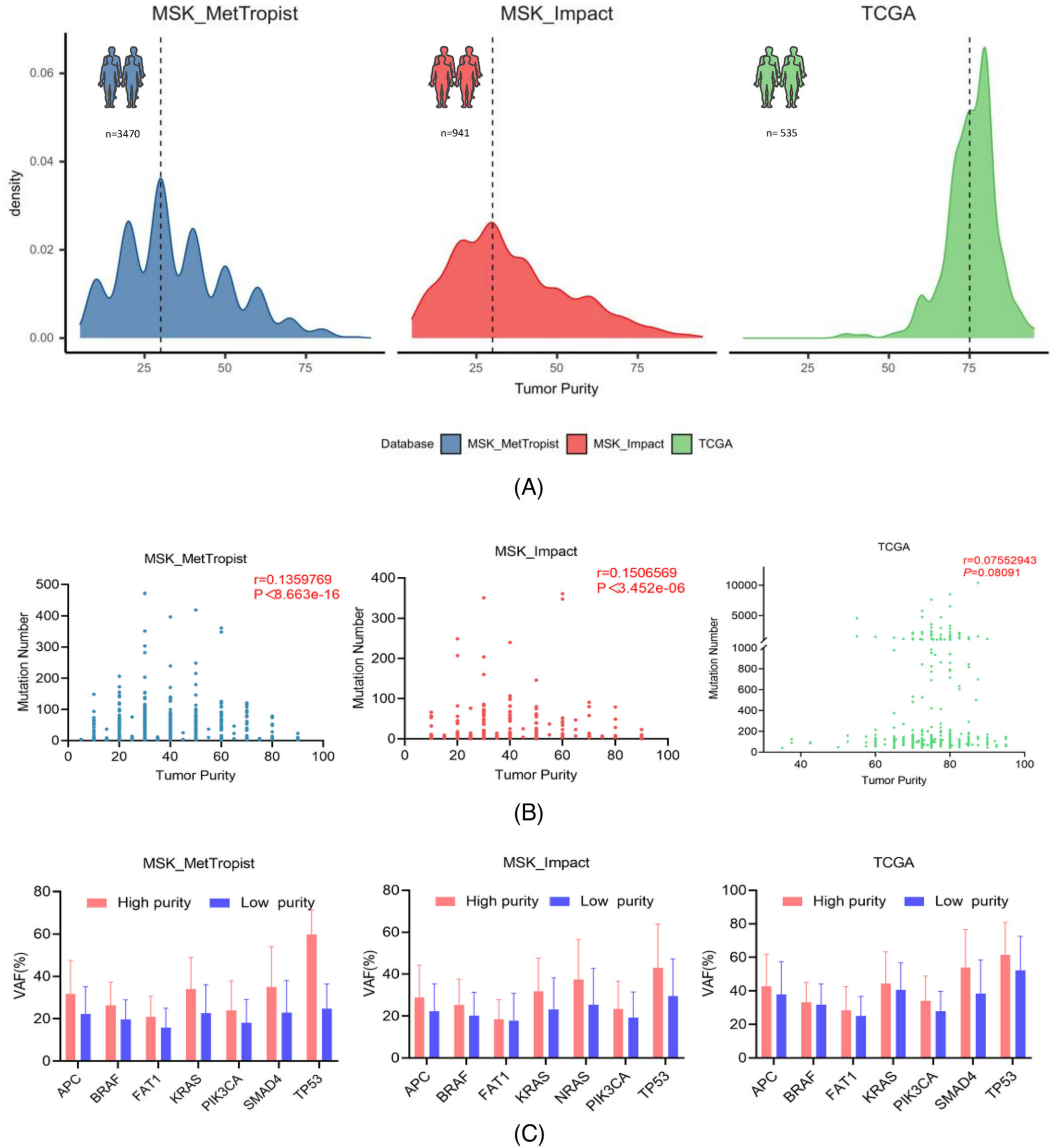
Influences of tumour purity on mutation detection in colorectal cancer (CRC) samples from public databases. (A) Sample size and distribution of purity in CRC cohorts from three public NGS datasets (MSK‐MetTropist, MSK‐Impact, The Cancer Genome Atlas [TCGA]). (B) Correlations (*p*, Spearman's correlation) between mutation number and corresponding tumour purity. (C) Comparison of variant allele frequencies (VAFs) of several mutated genes in three public CRC cohorts between high‐purity and low‐purity groups (cut‐off values of high‐purity and low‐purity groups were the median purity value in each cohort).

Initially, we identified the correlation between the genomic mutation profile called by MuTect2 and the corresponding tumour purity from three public datasets: The Cancer Genome Atlas (TCGA) (*n* = 535),^5^ MSK‐IMPACT (*n* = 941)^6^ and MSK‐MetTropist (*n* = 3470).^7^ Samples with low tumour purity were widespread in real‐world NGS datasets (Figure 1A). The quantity of mutations and tumour purity had a favourable correlation (Figure 1B). Similar results were found in TCGA called by MuSE, SomaticSniper and VarScan2 (Figure S1A). To verify the impact of tumour cell fraction on the variant allele frequency (VAF) of mutated genes, we classified the database samples into high‐fraction and low‐fraction groups based on the median tumour cell fraction. The VAF of majority of common hotspot variants in high tumour cell fraction samples displayed considerably higher or positive correlational trends than those in the low tumour cell fraction samples (Figure 1C). Similar results were also observed when the variants were called using MuTect2, MuSE and VarScan2 algorithms in TCGA (Figure S1B). These findings suggested that the number and VAF of variants may be significantly underestimated in low tumour cell fraction samples.

Then, we systematically evaluated the impact of tumour cell fraction on the fidelity of NGS with 30 surgical specimens using a targeted NGS platform including exon of 437 cancer‐associated genes and intron of 62 genes where fusion usually happens (1.53 Mb). The detailed clinicopathological parameters of the patient cohort are shown in Table S1. Paired serial‐sectioned samples after tumour purity assessment were alternately divided into precise‐sampling and routine‐sampling groups according to the sampling sequence. Precise scratching sampling for tumour‐specific tissue was performed in precise‐sampling groups so as to improve the tumour purity (Figure 2A) (Supplementary Method 1). The clinical–genomic features of 30 paired CRC samples with precision sampling and routine sampling are summarised in Figure 2B. A total of 250 mutations were private to the precise‐sampling group, 23 mutations were private to the routine‐sampling group, and 439 mutations were shared mutations (Figure S3). The distribution of variants under changes in tumour purity are shown in Figure 2C.

FIGURE 2Study design and clinicopathological and genomic characteristics of paired‐sample colorectal cancer (CRC) cohort. (A) Study design to evaluate tumour purity factors affecting sequencing results and minimise interference from intra‐tumoural heterogeneity. (B) Heatmap illustrating clinical and genomic data for the 30 paired‐sample CRCs. (C) The distribution of variants under changes in tumour purity. (D) Mutation numbers in routine‐sampling and corresponding precise‐sampling groups with different tumour purities. (E) Common mutation variant allele frequencies (VAFs) before and after precise sampling in relation to tumour purity. (F) Numbers and copy number increments of CNVs before and after precise sampling. (G) Effect of tumour purity on tumour mutational burden (TMB) estimates in precise and routine samples with different tumour purities.

**Figure:**
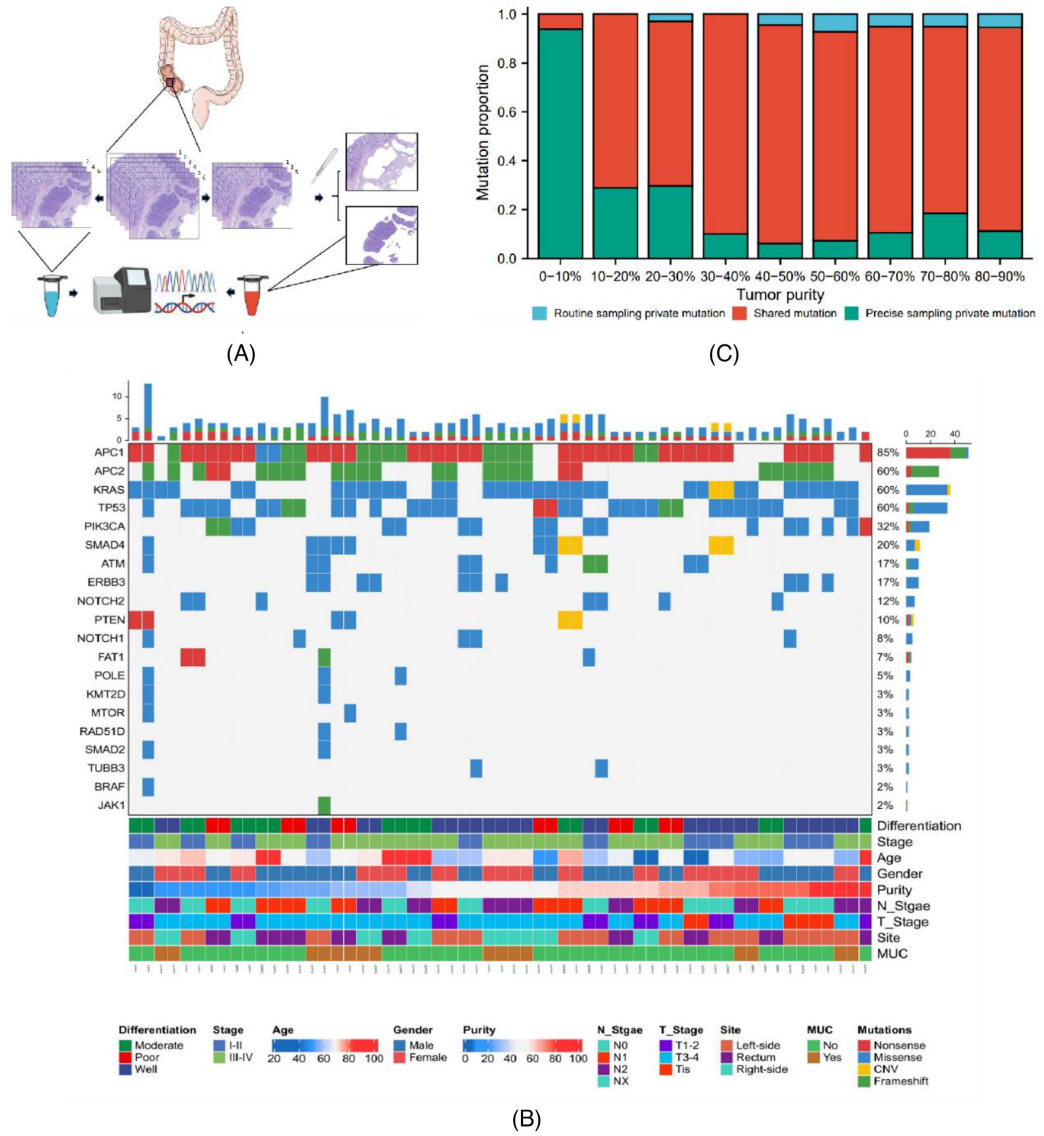

**Figure:**
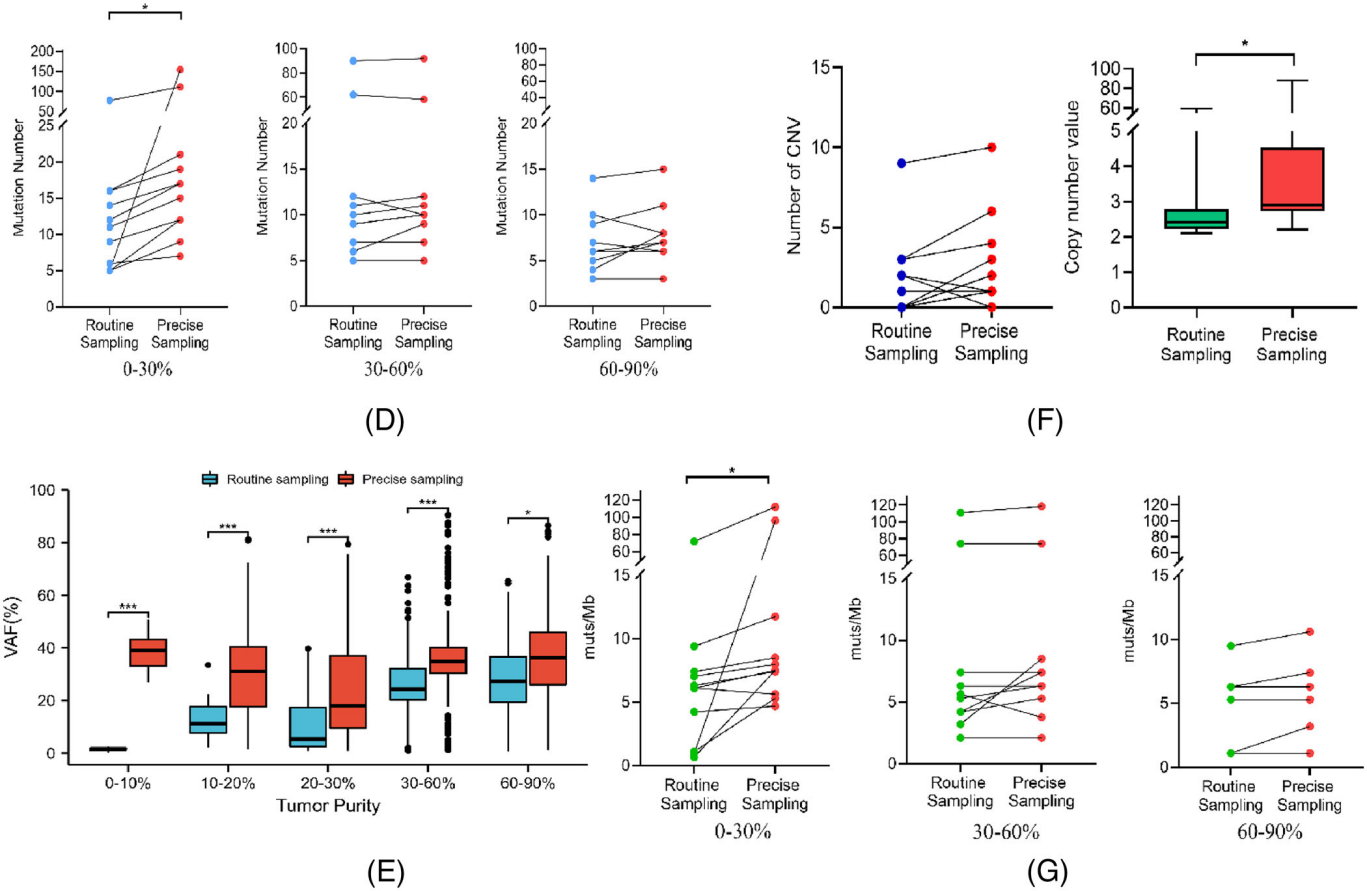

**FIGURE 3:**
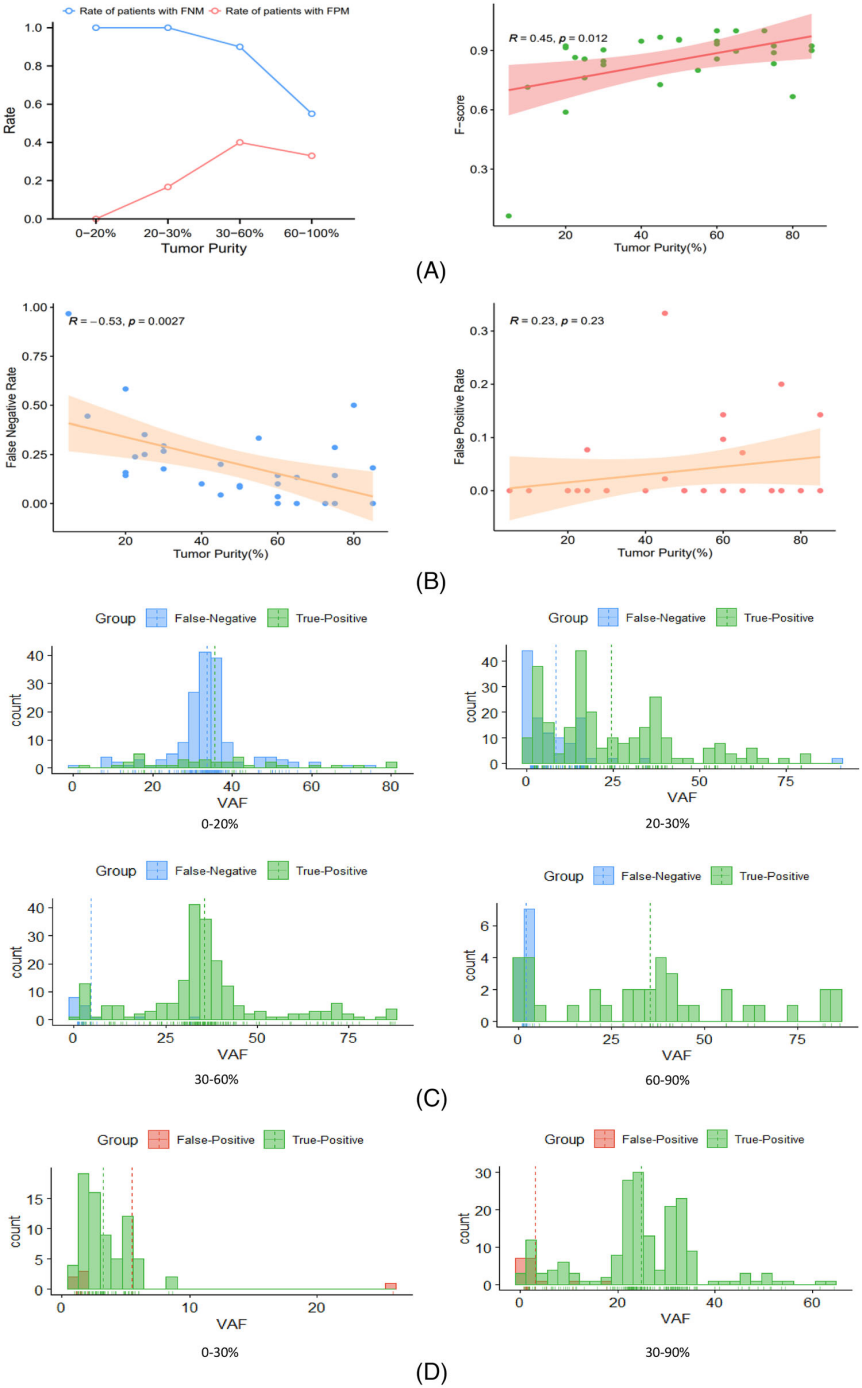
Threshold of tumour purity for mutation calling. (A) Prevalence of samples with different tumour purities containing false‐negative mutations (FNMs) and false‐positive mutations (FPMs) (left) and correlations between accuracy of mutation detection (*F*‐score) and tumour purity (right). (B) Correlations between false‐negative rate (left)/false‐positive rate (right) and tumour purity. (C) Mutation numbers and variant allele frequency (VAF) distributions of FNMs and true‐positive mutations in tumours with routine samples in different purity groups. (D) Mutation numbers and VAF distributions of FPMs and true‐positive mutations in tumours with routine samples in different purity groups.

The number of mutations after precise sampling was significantly increased in low tumour purity group (Figure 3D). The VAFs of common hotspot mutations were also considerably increased in precise‐sampling group (Figure 3E). There was an increasing trend in the number of genes with copy number variations (CNVs) after precise sampling, and the copy number values of genes in the precise‐sampling group changed obviously compared with those in the routine‐sampling group (Figure 3F). Tumour mutational burden was also underestimated in low‐purity sample group (Figure 3G). To further rule out an influence of pathological factors on mutation detection, we performed subgroup analyses according to location, staging and differentiation of tumour and similar outcomes were confirmed (Figure S4). These findings showed that pathological parameters had no effect on the bias influence of tumour purity on mutation detection.

We further identified the optimal tumour purity threshold for calling mutations, and we regarded precise‐sampling private mutations as false‐negative mutations (FNMs) and routine‐sampling private mutations as false‐positive mutations (FPMs). The proportion of patients with FNMs gradually reduced as tumour purity increased, while the proportion of patients with FPMs was relatively small and showed no correlation with tumour purity (Figure 3A). We evaluated the accuracy of mutation detection using the *F*‐score (Supplementary Method 2). The accuracy of mutation detection increased as the tumour purity increased (Figure 3A). We further investigated the reason for the poor accuracy of mutation detection in low tumour cell fraction samples by analysing the association between the false‐negative/‐positive rate and tumour purity. The false‐negative rate of the samples decreased as tumour purity increased, while the false‐positive rate was not significantly correlated with tumour purity (Figure 3B). The variants were then described and displayed based on their VAF. When the tumour purity was <30%, there were many FNMs with high‐VAF variants. The number of FNMs decreased significantly when the tumour purity was >30%, and most of these were low‐VAF variants (Figure 3C). In contrast, there was no connection between the quantity of FPMs and tumour cell fraction, and the detected FPMs were very low‐VAF variants (Figure 3D).

**FIGURE 4:**
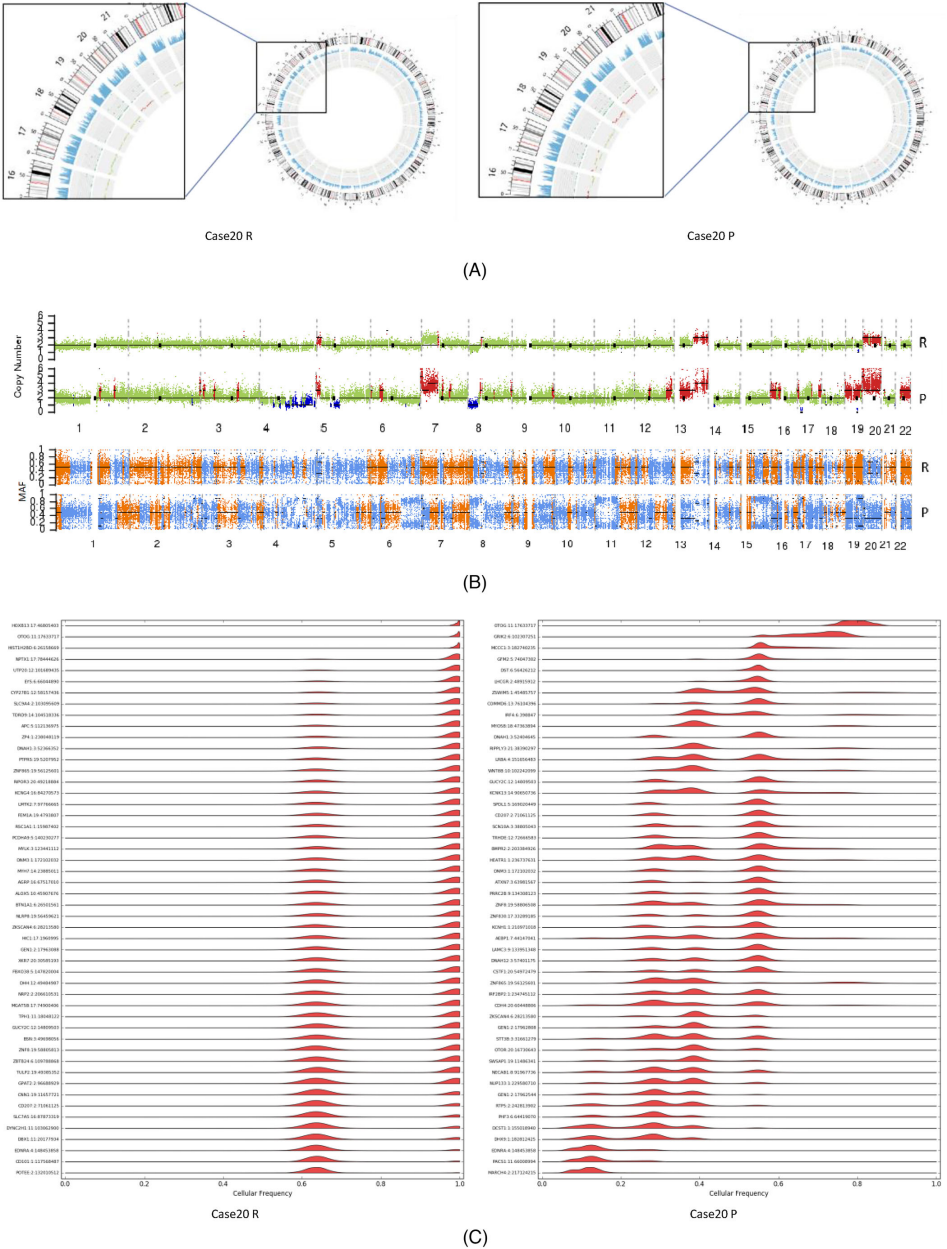
Influence of tumour purity on mutation calling by whole exome sequencing (WES). (A) Circos diagram of genomic mutation prevalence in relation to tumour purity. Left: routine sampling; right: precise sampling. Circle 1: sequencing coverage map; circle 2: sequencing coverage; circle 3: green dots represent density of SNVs and indels; circle 4: CNV results, red indicates increased copy number, blue indicates missing copy number and green indicates normal copy number. (B) Comparison of distributions of CNVs (above) and minor allele frequencies (MAFs) (below) in the genome between routine and corresponding precise samples in the whole exon. Red indicates increased copy number, blue indicates decreased copy number and green indicates no change in copy number. The following figure shows the distribution of MAFs. Loss of heterozygosity occurs when MAF is divided into 0 and 1. Orange indicates that AB allele distribution is consistent; blue indicates that AB allele distribution has preference. (C) Comparison of clone numbers between routine and precise sampling. Left: routine sampling, subclone number is 2; right: precise sampling, subclone number is 8.

Using case 20 as an example, we assessed the impact of tumour cell fraction. on mutation detection with whole exome sequencing (WES). The tumour purity of routine samples evaluated by pathologist and WES were 22.5% and 25%, respectively. The tumour purity was 100% after precise sampling. When compared to the routine sample, the precise sample had more genes with single nucleotide variants (SNVs) and indels (Figure 4A). The number and extent of CNVs that were amplified or deleted increased after precise sampling. The minor allele frequency (MAF) distribution preference and number of heterozygosity deletions were also underestimated in the low‐purity samples (Figure 4B). Due to many variants with low VAF were found, the number of subclones inferred after precise sampling was eight as opposed to two in routine sample (Figure 4C). Similar results were also observed in case 22 (Figure S5). Low tumour purity may affect the evaluation of mutation spectra and signatures, while cluster analysis with known mutation characteristics showed that differences in these factors did not affect the explanation of the carcinogenic mechanism (Figure S6). More drivers and target genes were detected after precise sampling (Figure S7).

In conclusion, we unveil that tumour purity acts as an independent and significant influencing factor and should be taken into consideration when evaluating genomic characterisation using NGS detection in CRC. Above 30% of tumour purity might be suitable for clinical applications in precision oncology and a higher tumour fraction could promote the accuracy of WES for assessing mutational and clonal landscapes.

## CONFLICT OF INTEREST STATEMENT

The authors declare they have no conflicts of interest.

## Supporting information



Supporting Information

Supporting Information
