# Supplementary material for: Tumour purity as an underlying key factor in tumour mutation detection in colorectal cancer
Source: Clin Transl Med. 2023 May 2;13(5):e1252. doi: 10.1002/ctm2.1252 (PMC10154864; doi:10.1002/ctm2.1252)
Supplement: Supplementary file 2 — Supporting Information [file CTM2-13-e1252-s002.docx]

**Extended Data**

Figure S1. Influences of tumor purity on mutation calling using three other mutation-calling algorithms in TCGA cohorts. (A) Correlations between numbers of mutations called by MuSE, SomaticSniper, and VarScan and the corresponding tumor purities of CRC samples in TCGA. (B) Comparison of VAFs of several common mutations called by three other mutation-calling algorithms (MuSE, SomaticSniper, and VarScan) between high-purity and low-purity groups in TCGA (cut-off value of tumor purity: 80%). (TCGA, The Cancer Genome Atlas; VAF, variant allele frequency; CRC, colorectal cancer).

Figure S2. Process of precise sampling. (A) Hematoxylin-eosin staining was performed to evaluate the tumor purity and determine the range of precise sampling. Landscape after Histogene dyeing (B) and after precise sampling (C).

Figure S3. Venn diagram showing numbers of mutations called using precise and routine sampling in 30 paired CRC samples.

Figure S4. Effects of tumor purity on common mutation VAFs and numbers in different clinicopathological subgroups. (A) Effects of tumor purity on mutation numbers in routine and corresponding precise samples within subgroups according to tumor stage, tumor location, and degree of differentiation. (B) Effects of tumor purity on common mutant VAFs in routine and corresponding precise samples within subgroups according to tumor stage, tumor location, and degree of differentiation.

Figure S5. Influence of tumor purity on mutation calling by WES. (A) Circos diagram of genomic mutation prevalence for different tumor purities. (Left: routine sampling; Right: precise sampling). (Circle 1: sequencing coverage map; Circle 2: sequencing coverage; Circle 3: green dots represent density of SNVs and indels; Circle 4: CNV results, red indicates increased copy number, blue indicates missing copy number, and green indicates normal copy number). (B) Comparison of distributions of CNVs (above) and MAF (below) in the genome between routine and corresponding precise sampling in the whole exon. Red indicates increased copy number, blue indicates decreased copy number, and green indicates no change in copy number. The following figure shows the distribution of MAFs. Loss of heterozygosity occurs when MAF is divided into 0 and 1. Orange indicates that AB allele distribution is consistent; blue indicates that AB allele distribution has preference. (C) Comparison of clone numbers between routine and precise sampling. (Left: routine sampling, subclone number is 3; Right: precise sampling, subclone number is 4).

Figure S6. Impact of tumor purity on mutation spectrum and mutation signature. (A) Distribution of mutation spectra in samples with routine and precise sampling. (B) Mutation signatures obtained by clustering 96 mutation types using the non-negative matrix factorization algorithm. (C) Impact of tumor purity on proportion of mutation signatures. (D) Cosine similarity heatmap of mutation signatures.

Figure S7. Landscape maps of driver mutations (A) and drug-targeted mutation (B) in samples before and after precise sampling
