# Supplementary material for: Tumour purity as an underlying key factor in tumour mutation detection in colorectal cancer
Source: Clin Transl Med. 2023 May 2;13(5):e1252. doi: 10.1002/ctm2.1252 (PMC10154864; doi:10.1002/ctm2.1252)
Supplement: Supplementary file 2 [file CTM2-13-e1252-s001.docx]

L10_P3	Case1	73	Female	Moderate	ⅡA	Left-side	20	No
L11_P3	Case2	67	Male	Moderate	Ⅰ	Left-side	5	No
L12_P3	Case3	68	Male	Poor	ⅣA	Rectum	20	No
L13_PB2	Case4	69	Male	Poor	ⅣC	Rectum	30	Yes
L41_P2	Case5	69	Male	Well	Ⅰ	Left-side	80	No
L18_P2	Case6	56	Female	Well	ⅢC	Left-side	75	Yes
L29_P2	Case7	70	Female	Well	ⅢB	Right-side	50	Yes
L20_P4	Case8	55	Male	Moderate	ⅢB	Rectum	75	No
L23_P1	Case9	70	Female	Moderate	Ⅰ	Right-side	20	No
L26_P1	Case10	75	Female	Moderate	ⅢC	Left-side	60	No
L29_L1	Case11	70	Female	Well	ⅢB	Right-side	10	Yes
L54_DP4	Case12	59	Male	Well	ⅡC	Left-side	45	No
L18_P3	Case13	56	Female	Well	ⅢC	Left-side	85	Yes
L29_P3	Case14	70	Female	Well	ⅢB	Right-side	50	Yes
L30_P1	Case15	58	Female	Well	ⅢA	Left-side	45	No
L33_P3	Case16	50	Male	Poor	ⅣC	Right-side	55	No
L34_P1	Case17	83	Female	Moderate	ⅣB	Rectum	30	No
L41_P1	Case18	69	Male	Well	Ⅰ	Left-side	75	No
L15_直P1	Case19	69	Male	Poor	ⅢB	Rectum	25	No
L43_P1	Case20	83	Male	Moderate	ⅢC	Rectum	22.5	No
L50_P1	Case21	68	Female	Well	Ⅰ	Left-side	72.5	No
L51_HP4	Case22	83	Male	Moderate	ⅢC	Right-side	40	No
O18_CC1	Case23	38	Female	Well	Ⅰ	Rectum	65	No
L54_DP2	Case24	59	Male	Well	ⅡC	Left-side	25	Yes
L51_RP4	Case25	83	Male	Moderate	ⅢC	Rectum	85	No
L58_P5	Case26	68	Male	Poor	ⅣB	Left-side	65	No
L61_P1	Case27	67	Male	Poor	ⅢC	Rectum	60	No
L62_P2	Case28	38	Female	Moderate	ⅢA	Right-side	60	No
L54_SP1	Case29	59	Male	Well	ⅡC	Left-side	60	No
L29_P1	Case30	70	Female	Well	ⅢB	Right-side	30	Yes
